# Research Staff Perspectives on Cancer Clinical Trials and Barriers to Recruitment: A Qualitative Research

**DOI:** 10.7759/cureus.17202

**Published:** 2021-08-15

**Authors:** Amany R Keruakous, Silas Day, Kenny Garcia-Ramiu, Melissa Yarbrough, Adam S Asch

**Affiliations:** 1 Hematology and Medical Oncology, Augusta University Medical College of Georgia, Augusta, USA; 2 Hematology and Medical Oncology, University of Oklahoma Health Sciences Center, Oklahoma City, USA

**Keywords:** cancer clinical trials, barriers to clinical trials enrollment, interventions to improve clinical trials accrual, quality improvement, research staff perspective

## Abstract

Background

Clinical trials are key elements of the processes that account for many of the recent advances in cancer care. Unfortunately, they are becoming more challenging to conduct. Furthermore, a large number of clinical trials in oncology close early due to poor accrual. To identify opportunities for continued improvement in clinical trial enrollment, we sought to identify the obstacles encountered by our clinical trial research staff in these activities.

Methods

This is a prospective qualitative study, using Grounded Theory Methodology that was concluded at Stephenson Cancer Center (SCC). SCC has been the lead accruer to National Cancer Institute-Lead Academic Participating Sites (NCI-LAPS) trials over the past three years, and in addition, fields investigator-initiated and industry-sponsored trials.

We conducted a survey of our research staff including all research nurses and disease site coordinators who participate in recruitment, screening, consenting, data collection, and compliance for interventional clinical trials. We then performed a follow-up meeting with our research coordinators to clarify responses. The study objectives were to highlight common barriers to recruiting adult cancer patients, encountered by research coordinators from all disease sites and to propose effective solutions to identified barriers.

Results

We are reporting our results of investigating barriers to clinical trials enrollment from a new perspective. The most commonly reported obstacles for clinical trials enrollment from our research staff's perspective were categorized into five themes: clinical trials protocol, communication barriers and cultural beliefs, financial barriers, patients' comorbidities and performance status, and physicians’ commitment.

Conclusions

Although assessing barriers encountered by clinical research staff is an infrequently used metric for improving clinical trial enrollment, it provides an important perspective in the field. Implementing interventions to improve clinical trial feasibility and accrual is critical to improving cancer care.

## Introduction

Clinical trials provide the foundation on which advances in cancer therapies are built, but so much stands in the way. While clinical trial treatment is considered on-guideline and may result in a lower financial burden to the patient, the national average of cancer patients accrued to clinical trials remains only 2-4% [1,2]. Poor accrual of cancer patients in cancer clinical trials has been a chronic problem in academia as well as community-based practices [3-6]. Furthermore, a large number of clinical trials in oncology close early due to poor accrual. In an analysis of clinical trials for adults registered on Clinicaltrials.gov, researchers found that about 20% of the trials fail to accrue for various reasons [7].

Reasons reported to be common obstacles for accrual in clinical trials are often investigated independently a) financial barriers [8], b) patient-reported factors [9,10], c) physician-related factors [11], d) barriers encountered in selected patient population [12-16], or e) selected disease sites [17,18]. Understanding the etiology of low patient accrual is necessary to improve participation in clinical trials. Researchers have attempted to identify the barriers to clinical trial enrollment by questioning individuals eligible for participation in clinical trials [9,19], physicians involved [10,20,21], or by exploring lessons from failed accruals [22].

The Stephenson Cancer Center, the only National Cancer Institute (NCI)-designated cancer center in the state of Oklahoma, has led accrual to NCI-Lead Academic Participating Sites (LAPS) trials over the past four years. The Stephenson Cancer Center also fields numerous investigator-initiated and industry-sponsored trials. In this study, we sought to identify common obstacles for trial accrual from a new perspective. We surveyed our clinical research staff-- specifically our research coordinators, who are the patient’s primary point of contact in the process of patients’ recruitment, screening, consenting, data collection, and compliance (Appendices).

The purpose of this study is to a) highlight common barriers to recruiting adult cancer patients encountered by research coordinators across multiple disease sites, and b) propose effective solutions to the identified barriers.

This article was previously published as a preprint article: Keruakous AR, Day S, Garcia-Ramiu KD, Yarbrough M, Asch AS: Research staff perspectives on cancer clinical trials and barriers to recruitment: a qualitative research. 18 March 2021, PREPRINT (Version 1) available at Research Square [https://doi.org/10.21203/rs.3.rs-322279/v1]

## Materials and methods

Study design and participants

This study is a prospective qualitative study, using Grounded Theory Methodology [23,24]. Given the lack of data on barriers encountered by research staff members during the patient's enrollment process in clinical trials, we thought that the qualitative method is appropriate for the study as we are exploring a new perspective by using inductive reasoning starting with data collection and interpretation to construct a theory.

The grounded theory method was felt to be fit for the research question as it addresses their experience and interactions with the patients to identify and categorize barriers to clinical trial enrollment to develop a theory from the analysis grounded in the data provided.

To achieve purposive sampling, we choose participants who are working in a direct relationship with cancer patients from all disease sites and serving a wide diversity of patients. Thus, we focused on the research staff involved in interventional clinical trials in this manner. Participants for this qualitative study were approached via email as we distributed a formal survey that asked open-ended questions to procure frequently encountered obstacles that survey participants meet during their daily interactions with our patients

The number of participants was determined by reaching a point of data saturation (i.e. no more new themes emerging); we then performed follow-up meetings with the respondents using semi-structured interviews which were guided by topic guides and adjusted based on the initial analysis or their email responses which were helpful to clarify and add richness to the data collected.

Data collection

Data were collected through a survey that was sent via e-mail to describe the following: 1) the disease site they are primarily involved, 2) what is their role in clinical trials (data management, compliance, etc.), 3) what percentage of screened patients decline participation in the clinical trial, 4) what are the most common reasons that patients decline participation, 5) what are the main obstacles to enrollment, and 6) additional comments (where they had the opportunity to describe any additional information and suggestions, in addition to the semi-structured interviews).

Data analysis

De-identified surveys and follow-up transcribed meetings were collected in one software. Data were categorized into codes creating a codebook to ensure inter-coding agreement. Emerging codes were then discussed and agreed upon by team members. Emerging codes led to themes. The process of defining and refining the themes was continuous throughout the data analysis. Selected research coordinators were asked to review the data collected and the manuscript to ensure confirmability.

## Results

Sample

Twenty-one surveys were received from research coordinators at different disease sites. Of these 21, six surveys were from gynecologic malignancies, five from breast oncology, three from lung and head/neck malignancies, four from malignant hematology, two from gastrointestinal malignancies, and one from genitourinary malignancies.

Themes

Five main themes important to clinical trial enrollment were identified: clinical trials protocol, communication barriers and cultural beliefs, financial barriers, patients' comorbidities and performance status, and physicians’ commitment. Below we will describe the themes.

Clinical Trial Protocol

A common obstacle encountered by our research coordinators pertains to the clinical trial schema and protocol requirements. Participants frequently reported that the inclusion and exclusion criteria are often overly strict. Furthermore, it was noted that patients commonly tend to refuse to participate in clinical trials if additional biopsies are required.

Also, frequent laboratory testing and office visits often drive patients away from participation in clinical trials because of their concern of affecting their work schedule and increase the time off work to participate in such activities, especially if they live at a distance from the treating institution. Patients prefer a more flexible approach to treat their condition and preserve their quality of life.

Communication Barrier and Cultural Beliefs

Frequently reported, the communication barrier has been one of the difficult obstacles to overcome. If the patient is speaking a non-English language, commonly Spanish, that often excludes them from participating in clinical trials. The absence of language-appropriate consent or the need to requesting institutional review board (IRB) approval of Spanish informed consent delays the enrollment procedure. The urgency to start the treatment makes waiting to participate in the clinical trial an inferior option for the majority of our patients. 

Cultural beliefs linked to the fear of receiving an investigational drug or a placebo are commonly encountered. Also, the common conception of being treated as a “lab rat” while participating in a clinical trial usually exists as a barrier for accrual. These cultural beliefs and misconceptions have also led to the underrepresentation of racial minorities in cancer clinical trials.

Financial Burden

Patients refuse to participate in the clinical trial due to the concern of the additional financial implications that might fall with the additional office visits and laboratory testing, let alone arranging transportation for those visits may create an added barrier. Additionally, the legitimate concern of having extra days off work to accommodate the clinical trial office visits and laboratory testing, adding yet another layer of financial burden.

Patients’ Status

Disease burden affects the patients’ general performance status and subsequently excludes them from clinical trials. This barrier can often be counterintuitive to the patient’s ultimate well-being. As performance status limitations are often directly or indirectly related to their underlying malignancy, and in such settings, treating the underlying malignancy serves to improve their energy level and performance status. Also, comorbidities remain a common exclusion criterion. 

Physician Commitment

Explaining the science behind the investigational agent, its mechanism of action, and the study hypothesis as well as disclosing benefit/risk balance while incorporating the financial burden and time commitment is crucial for clinical trial screening. Due to the level of confidence and trust built between the primary oncologist and the patients/their families, the clinical trial is best introduced and explained by the treating physician, which requires physician enthusiasm and commitment as well as open communication with the research team.

Clinical Trials Challenges in the Midst of a Pandemic

During the COVID-19 pandemic, our research staff noted continued interest in clinical trials. However, patients express concerns over multiple in-person visits required on trial. Lack of education surrounding risks of admission vs risks of immunosuppression needs to be addressed. Also, screen failures have become more prominent throughout the pandemic as patients delay medical care until the situation is urgent. This leads to a worsened disease burden, worsened performance status, organ dysfunction which disqualifies them from participation in many clinical trials.

Illustrative quotes are provided in Table 1. 

**Table 1:** Selected quotes illustrating themes and codes

Themes	Codes	Quoted responses
Clinical trial protocol	- Frequent laboratory testing and office visits. -Treatment schedule and time commitment. -Strict inclusion/exclusion criteria. -Additional biopsy requirements.	“Patients often find that: a) Office visits are too much b) Laboratory tests are too frequent in between office visits c) Treatment schedules interfere with their work schedule hence affecting the quality of life and preclude financial burden" “Patients live too far away and would prefer a less regimented treatment which provides more logistical wiggle room” “Strict Inclusion/Exclusion has been the main barrier to enrollment” “Mandatory tissue requirements of certain specifications often make patients reluctant to participate”
Communication barrier and Cultural beliefs	-Language barrier -Refusal to be on an investigated subject -Concerns about the risks with an investigational agent	“Patients often are not willing to try investigational drugs” “Patients decline is due to the fear of being experimented on” "Patients have the cultural belief that if they are on a clinical trial, they are being treated as a guinea pig or a lab rat and refuse to listen about what the trial entails" “Patients do not want to take the risk of being on placebo arm and would rather standard treatment” “whenever we have not an English speaker patient, we have difficulty enrolling them on a clinical trial even if they are cooperative due to lack of foreign language informed consent”
Financial barriers	- Financial burden of additional laboratory testing and office visits. -Burden of extra days off to accommodate the trial protocol	“A lot of times, patients have expectations that all treatments/procedures while on the study are provided by the study, they get quite anxious with costs of the additional laboratory testing and the transportations needed” “The fear of missing extra days off work to follow the study treatment schedule and office visits”
Patients’ status	-Performance status -Comorbidities	“Most of the clinical trials limit inclusion criteria based on performance status which might be affected due to the disease burden” “Declining physical condition due to disease burden excludes patients” “Comorbidities is often what excludes patients from participating in clinical trials”
Physician commitment	-Building a foundation of confidence. -Appropriately introducing the research team.	“The physicians doing a good job of rationalizing why patients are ideal candidates for a clinical trial prior to the research nurse/coordinator going in for the consent” “The physician is the person our patient most relies on to make informed decisions on their healthcare” “Building a foundation of confidence for the clinical trial and the research”
Clinical trials challenges in the midst of a pandemic	-Delays to seek medical attention leads to more screening failures -Continued interest but lacking education -Implementing telemedicine visits	“...have not seen a decreased interest in clinical trial enrollment throughout the pandemic” “Clinical trial infrastructure has been hit during the pandemic” “Patients seem to be more concerned about contracting COVID at the hospital than in the community” “Patients are not seeking healthcare as soon as they normally would sue to the pandemic” “More screen failures as patients’ diseases have progressed to the point their performance status or hepatic function is no longer compatible with the clinical trial” “Accommodate some telemedicine visits for trial patients as well as continue to provide them a safe face to face visit as well with universal masking, screening at every entrance and limited visitors” “Re-evaluate what procedures and visits in clinical trials are “unnecessary” in hopes of minimizing the exposure risk for our patients”

## Discussion

Identifying common obstacles for clinical trial enrollment is a key to improving the clinical trial accrual rate, therefore improving the advancement in cancer treatments [25]. We explored this aspect from our research staff perspective. We aimed to identify barriers to clinical trials accrual and propose potential interventions in future trials protocols that would improve the accrual rates and facilitate patients’ enrollment and commitment in clinical trials.

The complexity of clinical trials protocol is a reported obstacle by our research coordinators and other studies [26]. Clinical trial protocols that include simple schema, minimizing office visits, and frequency of laboratory testing would be more feasible for patients to participate in clinical trials. If frequent laboratory testing is necessary for investigational drug monitoring, utilizing home health care services for eligible patients to perform blood draws for required laboratory testing, as well as utilizing virtual care visits to follow up on patients’ tolerance; would facilitate patients’ commitment to clinical trials and eliminate the financial burden of frequent transportation.

Although industry-sponsored clinical trials offer travel reimbursement programs, having this implemented universally for all trials would also aid in recruitment. Utilizing social workers' services to help with transportation aids that are offered by patients’ primary insurance should also be considered. Other tools to improve patients’ accrual are to broaden the eligibility criteria considering the patients’ comorbidities and performance status, as well as minimize additional biopsies required for accrual.

Cultural beliefs are often non-modifiable; however, cultural influences must be considered when recruiting for clinical trials. Encouraging the physicians and research staff to use basic or simple terms in explaining the clinical trials and their purpose while simultaneously addressing patients’ concerns, and emphasizing that participating in a clinical trial does not entail being an experimental subject [27,28]. It is noted that despite having a high education level, 75% of clinical trials participants did not have enough basic research knowledge to make a decision about joining a cancer clinical trial [29]. Community organizations educating the public about cancer research may be the key to enhancing patient enrollment [30].

A language barrier is encountered by one-third of cancer patients as determined by Surveillance, Epidemiology, and End Results (SEER) database statistics review 2000-2017. The majority of these patients are Spanish speaking, therefore having a Spanish informed consent form with every clinical trial protocol suffices as a measure that would efficiently facilitate non-English speakers to participate in clinical trials. At the same time, it demonstrates to that specific patient population that their participation in clinical trials is important in furthering cancer research.

Finally, delivering the best care for cancer patients in general as well as cancer clinical trials has been challenging through the COVID-19 pandemic. Balancing the risks of treatment procedures, its potential complications, and exposure to COVID-19, using the ultimate safety measures following the US Centers for Disease Control and Prevention (CDC) guidelines is crucial. Re-evaluating the infrastructure of clinical trial operations by revising procedures and modifying required visits to minimize exposure should be widely implemented.

Utilizing a qualitative research method using open-ended questions could limit the study, as participants have more control over the content of the data collected, making the data not verifiable objectively against the scenarios stated by the respondents. Simultaneously, this adds another value as it emphasizes patterns that can be hard to see, due to the breadth of emotions, reactions, and responses from study participants.

The research is based more on the opinion and judgment of our research staff rather than on measurable outcomes, and it does not investigate causality, but it does provide indications of possible directions for academic and sponsored clinical trials teams to improve the feasibility of clinical trials and bolster the potential accrual efforts that could be implemented. 

## Conclusions

Assessing barriers encountered by clinical research staff is an infrequently used metric for improving clinical trial enrollment, but provides an important perspective. This qualitative study is reporting a new perspective for barriers to clinical trials accrual encountered by our research staff coordinators. The main five themes reported by our research staff as commonly encountered obstacles to clinical trails enrollment are: the complexity of clinical trials protocol, communication barriers and cultural beliefs, financial barriers, patients’ comorbidities and performance status, and physicians’ commitment.

In our study, some obstacles are inherent in our patient populations, others appear to be actionable. We proposed possible measures that could be implemented into clinical trial protocols to facilitate patients’ accrual. The complexity of clinical trial protocols and the increasingly strict inclusion/exclusion criteria are issues that will require consideration and action at the level of the cooperative groups and industry. Additionally, accommodating communications and cultural barriers with increasing institutional and physicians' commitment could overcome negative bias regarding clinical trials, and enhance recruiting underrepresented minorities.

## Appendices

Research staff survey

Dear Research staff,

Please share your opinion on obstacles to clinical trial enrollment.

I’m trying to track factors that potentially affect patient enrollment into cancer clinical trials; and as you’re all intimately involved with screening and consenting patients here at SCC, I would be very grateful to get your feedback.  

1.      Is there a disease site in which you are primarily involved?

2.      What’s your role in clinical trials (nurse, coordinator, data, compliance, etc)? 

3.      What are the most common reasons that patients decline participation? 

4.      What are the main obstacles to enrollment (language, finances, overly strict inclusion/exclusion criteria, cultural issues, etc.) 

5.      Any additional comments on the process would also be appreciated.   

Thanks to all of you for your time and your help to improve patients’ enrollment in cancer clinical trials.
